# Single crystal spectroscopy and multiple structures from one crystal (MSOX) define catalysis in copper nitrite reductases

**DOI:** 10.1073/pnas.2205664119

**Published:** 2022-07-21

**Authors:** Samuel L. Rose, Seiki Baba, Hideo Okumura, Svetlana V. Antonyuk, Daisuke Sasaki, Tobias M. Hedison, Muralidharan Shanmugam, Derren J. Heyes, Nigel S. Scrutton, Takashi Kumasaka, Takehiko Tosha, Robert R. Eady, Masaki Yamamoto, S. Samar Hasnain

**Affiliations:** ^a^Molecular Biophysics Group, Life Sciences Building, Institute of Systems, Molecular and Integrative Biology, Faculty of Health and Life Sciences, University of Liverpool, Liverpool, L69 7ZB, United Kingdom;; ^b^Protein Crystal Analysis Division, Japan Synchrotron Radiation Research Institute, Hyogo, 679-5198, Japan;; ^c^Manchester Institute of Biotechnology and School of Chemistry, Faculty of Science and Engineering, The University of Manchester, Manchester M1 7DN, United Kingdom;; ^d^RIKEN SPring-8 Center, Sayo, Hyogo, 679-5148, Japan

**Keywords:** catalysis, electron transfer, metalloproteins, reaction intermediates, substrate utilization

## Abstract

Many enzymes utilize redox-coupled centers for performing catalysis where these centers are used to control and regulate the transfer of electrons required for catalysis, whose untimely delivery can lead to a state incapable of binding the substrate, i.e., a dead-end enzyme. Copper nitrite reductases (CuNiRs), which catalyze the reduction of nitrite to nitric oxide (NO), have proven to be a good model system for studying these complex processes including proton-coupled electron transfer (ET) and their orchestration for substrate binding/utilization. Recently, a two-domain CuNiR from a *Rhizobia* species (*Br*^2D^NiR) has been discovered with a substantially lower enzymatic activity where the catalytic type-2 Cu (T2Cu) site is occupied by two water molecules requiring their displacement for the substrate nitrite to bind. Single crystal spectroscopy combined with MSOX (multiple structures from one crystal) for both the as-isolated and nitrite-soaked crystals clearly demonstrate that inter-Cu ET within the coupled T1Cu-T2Cu redox system is heavily gated. Laser-flash photolysis and optical spectroscopy showed rapid ET from photoexcited NADH to the T1Cu center but little or no inter-Cu ET in the absence of nitrite. Furthermore, incomplete reoxidation of the T1Cu site (∼20% electrons transferred) was observed in the presence of nitrite, consistent with a slow formation of NO species in the serial structures of the MSOX movie obtained from the nitrite-soaked crystal, which is likely to be responsible for the lower activity of this CuNiR. Our approach is of direct relevance for studying redox reactions in a wide range of biological systems including metalloproteins that make up at least 30% of all proteins.

## Significance

X-rays used to collect crystallographic data can change the redox states of transition metals utilized by many biological systems including metalloproteins. This disadvantage has been harnessed to drive a complex chemical reaction requiring the delivery of an electron to the active site and recording the structural changes accompanying catalysis, providing a real-time structural movie of an enzymatic reaction, which has been a dream of enzymologists for decades. By coupling the multiple-structures from one crystal technique with single-crystal and solution optical spectroscopy, we show that the electron transfer between the electron accepting type-1 Cu and catalytic type-2 Cu redox centers is gated in a recently characterized copper nitrite reductase. This combined structural/spectroscopic approach is applicable to many complex redox biological systems.

Redox reactions are an essential component in a wide range of biological systems, most notably in respiration (1) and photosynthesis (2). These critical reactions are often performed by metal-containing systems including metalloproteins that form a large portion of the protein kingdom. It is estimated that one-third of all proteins in nature require metals to perform their biological roles and nearly one-half of all enzymes must associate with a particular metal to function (3, 4). These metal ions can be either a single atom or combined with other atoms to form part of a cluster, playing a variety of life sustaining roles in the bacterial, plant, and animal kingdoms. They exploit the oxidation states of metals to perform redox cycling during catalysis and include metalloenzymes such as cytochrome *c* oxidase, hydrogenases, nitrogenases, and nitrite reductases where catalysis involves the controlled delivery of electrons and protons to the active site for the utilization of chemical substrates. Most of these systems utilize multiple redox centers among which these catalytic events are often coordinated, coupled, and orchestrated by structural signals that remain poorly understood. The redox-active metalloenzyme copper nitrite reductase (CuNiR) has become a good model system for studying these complex processes in biological systems with coupled redox centers due to their amenability to spectroscopic, fast kinetic, and advanced structural approaches capable of providing a movie of catalytic reaction activated by electron transfer (ET) in crystallo between the coupled centers and its utilization for the conversion of substrate providing information on reaction intermediates, some of which may be transitory (5–9).

CuNiRs catalyze the conversion of nitrite to gaseous nitric oxide (NO_2_^−^ + 2H^+^ + e^−^ → NO + H_2_O) in the first committed step of the denitrification pathway. This highly conserved family of enzymes is widespread in nature (10) and is of major importance in several pathways in the biogeochemical nitrogen cycle (11, 12). These homo-trimeric proteins contain two types of redox Cu center per monomer, as follows: an electron accepting type-1 Cu (T1Cu) site, which receives an electron from a physiological redox partner (cytochrome *c* or pseudoazurin/azurin), and a catalytic type-2 Cu (T2Cu) site. The T1Cu site is located near the top of the monomer and is responsible for giving rise to the color of the enzyme (blue or green) in its oxidized [Cu^II^] state, depending on subtle differences in the immediate coordination chemistry of the site. The T2Cu is located within the interface of two adjacent monomers and is responsible for the binding of nitrite and its catalysis. The two redox Cu centers are coupled via the neighboring Cys-His residues that form a conserved hard-wired 12.6-Å ET bridge. When T2Cu is in the oxidized substrate-free [Cu^II^] state, with a Cu^II^-(His)_3_-H_2_O coordination, the difference between the reduction potentials of the two Cu sites are small or energetically unfavorable to allow ET from T1Cu to T2Cu. Displacement of the coordinated water by nitrite increases the reduction potential of T2Cu and promotes inter-Cu ET, an event heavily gated by the provision of protons from two conserved residues (His_CAT_ and Asp_CAT_) in the T2Cu pocket to the substrate (5, 13). In the CuNiR of *Achromobacter xylosoxidans* (*Ax*NiR), laser flash photolysis had shown the rate of inter-Cu ET to be the same as the rate of proton uptake, providing clear evidence for proton-coupled ET (PCET). Following the reduction of nitrite to nitric oxide (NO), the product dissociates from the T2Cu site and water rebinds to return to the resting T2Cu^II^ state. A delicate and ordered mechanism ensures a “dead-end” inactive species is not formed where the T2Cu redox center is in a reduced [Cu^I^] state with the water ligand disociated, that is then unable to bind the substrate (14, 15). This mechanism for CuNiR is supported by spectroscopic, kinetic, mutagenesis, and structural studies primarily from the well-studied blue *Ax*NiR and the green CuNiRs of *Achromobacter cycloclastes* (*Ac*NiR) and *Alcaligenes faecalis* (AfNiR) (13–18).

Biological systems containing redox centers, including metalloproteins such as CuNiR, are prone to reduction from X-ray sources during data collection (19, 20) due to solvated electrons within the crystal being produced from radiolysis of waters that can rapidly reduce their primary redox centers, e.g., T1Cu in CuNiRs is reduced from Cu^II^ to Cu^I^ redox state but the T2Cu remains oxidized (17). This phenomenon has been exploited to initiate enzyme turnover to generate reaction intermediates through redox driven catalysis (21). We have developed the MSOX (multiple structures from one crystal) approach that enables the collection of several structures from the same spot in a crystal (6–8). This approach has been applied successfully to *Ac*NiR at several temperatures to provide structural movies of the catalytic reaction in nitrite-soaked crystals. However, these studies have not provided any information on the redox state of metal centers during catalysis in the crystals.

A two-domain blue CuNiR from a *Rhizobia* species (*Br*^2D^NiR) has recently been structurally characterized showing an unusual oxidized T2Cu^II^-(His)_3_-(H_2_O)_2_ coordination site, requiring the displacement of two water molecules by the substrate (22, 23) instead of a single water molecule in a prototypic CuNiR (15, 24). The recent availability of highly diffracting crystals of *Br*^2D^NiR and the ability to achieve full occupancy of nitrite at the T2Cu site in nitrite-soaked crystals (22) prompted us to utilize the MSOX approach to probe structural changes during enzyme turnover. MSOX of as-isolated crystals of *Br*^2D^NiR was combined with the recently commissioned on-line single crystal optical spectroscopy at SPring-8 BL26B1, enabling the redox status of the optically visible T1Cu to be monitored during serially recorded multiple structures and reporting on the structural changes at the catalytic T2Cu site for a CuNiR in the resting state. This has enabled us to provide a spectroscopically validated MSOX movie of an as-isolated CuNiR. We have combined detailed structural observations recorded in these MSOX movies and single crystal spectroscopy with solution measurements of reduction potentials and inter-Cu ET using laser-flash photolysis. We provide unambiguous evidence for a strong gating of ET between the coupled redox centers that is removed in the presence of the substrate. We demonstrate that the experimental approach (marrying single crystal spectroscopy/MSOX with solution data) utilized here is powerful in dissecting complex redox reactions and suggest the approach should be applicable to many complex redox systems in biology.

## Results

### Combined Single Crystal Spectroscopy and MSOX Crystallography of Substrate-free *Br*^2D^NiR.

**Fig. 1.**
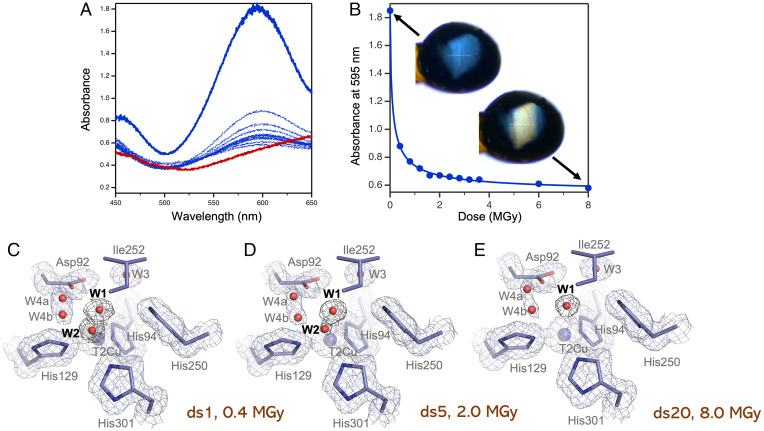
T2Cu site during the MSOX series of *Br*^2D^NiR in an as-isolated state and optical spectra of the *Br*^2D^NiR crystal for the duration of MSOX movie. (*A*) Optical spectra for a single crystal of blue *Br*^2D^NiR at 100 K. A peak at 460 nm and large peak at 595 nm prior to X-ray exposure is characteristic of a blue CuNiR with oxidized T1Cu^II^ (solid blue line). Exposure to 0.4-MGy X-rays results (corresponding to DS1, *C*) in a complete loss of the peak at 460 nm and a substantial reduction of the peak at 595 nm (first dotted blue line). Further exposure to 0.4-MGy X-rays per dataset up to a total dose of 8 MGy (corresponding to DS20, *E*) results in the sequential loss of the 595-nm peak (dotted blue lines). Optical spectra for a single colorless dithionite-reduced crystal prior to X-ray exposure is shown and is characteristic of reduced T1Cu^I^ (solid red line). (*B*) Dose-dependent reduction of the peak at 595 in *A* and color change of crystal from intense blue (prior to X-ray exposure) to colorless (after a final total dose of 8 MGy). (*C*) The T2Cu site after first exposure to 0.4-MGy X-rays (DS1) showing two full occupancy waters (W1 and W2) coordinated to T2Cu, Asp92 in proximal position and two positions of a channel water (W4). Ile252 and His250 show no changes. (*D*) The T2Cu site in DS5, after exposure to 2-MGy X-rays, shows only partial density for W2 consistent with its gradual loss, while W1 still has full occupancy. No other changes are seen. (*E*) The T2Cu site in the final dataset (DS20), after exposure to 8-MGy X-rays, showing W2 completely disappeared and W1 still had full occupancy. No other changes are seen. 2F_o_ − F_c_ electron density maps for residues and waters are contoured at 1σ level. T2Cu is shown as a blue sphere. Occupancy of T2Cu is similar to T1Cu. Both are judged to be >0.9 by comparison of their B-factors to their coordinating protein ligands.

Online optical single crystal spectroscopy has been combined with the MSOX X-ray crystallography approach using a single blue *Br*^2D^NiR crystal maintained at 100 K in the as-isolated state (i.e., in absence of nitrite). Optical spectroscopy prior to X-ray irradiation confirmed that the T1Cu site was in its oxidized [Cu^II^] state, as expected from studies with other blue CuNiRs (e.g., *Ax*NiR) (Fig. 1*A*). A total of 20 crystallographic datasets were obtained from the same spot of the *Br*^2D^NiR crystal with a dose of 0.4 MGy per dataset (estimated by RADDOSE-3D(25)). Following exposure to the first dose of X-rays, the optical spectrum showed a clear loss of the optical absorption peak at ∼460 nm and a substantial reduction of the peak at ∼595 nm (Fig. 1*A* and *B*). Each consecutive crystallographic dataset thereafter showed a sequential loss of the optical absorption peak at ∼595 nm with the crystal also changing color from intense blue to colorless (Fig. 1*A* and *B*). After 20 datasets and a total dose of 8 MGy, the spectrum demonstrated that the T1Cu site had converted to a reduced [Cu^I^] state in the crystal. A colorless dithionite-reduced crystal with optical spectroscopy measured before X-rays is shown for comparison (Fig. 1*A*). A crystallographic MSOX movie of serial structures to a resolution of 1.35 Å while the optical spectra were reporting on the redox status of T1Cu allowed us to define structural changes resulting from the T1Cu radiation-induced chemistry throughout the structure including the catalytic T2Cu site (*SI Appendix*, Movie S1). Data collection and refinement statistics are provided for dataset 1 (DS1), DS5, and DS20 in Table 1 as representatives of the data quality and refinement for the individual structures. The first structure showed two full occupancy water molecules (W1 and W2) coordinated to T2Cu as was previously observed for *Br*^2D^NiR (22) (Fig. 1*C*), with W1 more closely coordinated. Two conformations of the catalytic Asp residue (Asp92_CAT_) in its proximal and distorted position are also seen with two conformations of channel water (W4). Following a sequential reduction of T1Cu, the second T2Cu water (W2) begins to disappear (Fig. 1*D*), and eventually, only a single water (W1) is left coordinated to T2Cu in a perfect tetrahedral arrangement. This occurs by dataset 7 (2.8-MGy dose) and is reminiscent of the T2Cu^II^ oxidized state in other CuNiRs where only a single water has been observed. The single water remains present thereafter for the rest of the MSOX series (8 MGy) with only slight movement from a perfect tetrahedral arrangement being observed (Fig. 1*E*). There is no indication of Ile252 flipping throughout the series, which can only occur when T2Cu is Cu(His)_3_ tricoordinated and is devoid of a ligand. In all of the structures, no sign of rotation is seen in the catalytic His residue (His250_CAT_) with the residue forming a strong catalytic bridge with Asp92 via the bridging water (W3) throughout and remaining hydrogen bonded to Glu274. This MSOX series of as isolated *Br^2D^*NiR, coupled with optical spectroscopy, confirms that despite a full reduction of T1Cu and W2 burn-off, T2Cu remains coordinated to the closely coordinated water W1, similar to the resting [Cu^II^] state of other well-studied two-domain CuNiRs, despite significant exposure (8 MGys) to X-rays.

**Table 1.** Crystallographic data collection and refinement statistics

	***Br*^2D^NiR as-isolated MSOX DS1**	***Br*^2D^NiR as-isolated MSOX DS5**	***Br*^2D^NiR as-isolated MSOX DS20**	***Br*^2D^NiR nitrite-bound MSOX DS1**	***Br*^2D^NiR nitrite-bound MSOX DS8**	***Br*^2D^NiR nitrite-bound MSOX DS17**	***Br*^2D^NiR nitrite-bound MSOX DS25**	***Br*^2D^NiR nitrite-bound MSOX DS38**	***Br*^2D^NiR nitrite-bound MSOX DS65**
Data collection									
Space group	*P*2_1_3	*P*2_1_3	*P*2_1_3	*P*2_1_3	*P*2_1_3	*P*2_1_3	*P*2_1_3	*P*2_1_3	*P*2_1_3
Total dose (MGy)	0.4	2.0	8.0	0.8	6.4	13.6	20.0	30.4	52.0
Cell dimensions									
a = b=c (Å)	107.21	107.25	107.37	106.72	106.88	106.99	107.04	107.10	107.18
Resolution (Å)	47.95–1.35 (1.37–1.35)*	47.96–1.35 (1.37–1.35)*	48.02–1.35 (1.37–1.35)*	75.47–1.19 (1.21–1.19)*	75.58–1.17 (1.19–1.17)*	75.65–1.25 (1.27–1.25)*	75.69–1.32 (1.34–1.32)*	75.73–1.40 (1.42–1.40)*	75.78–1.54 (1.57–1.54)*
Unique reflections	88,206 (3,776)	88,290 (3,917)	88,590 (3,756)	128,957 (6,269)	135,725 (6,371)	112,299 (5,613)	95,644 (4,716)	80,496 (2,992)	60,762 (3,027)
Redundancy	5.7 (2.3)	5.7 (2.3)	5.7 (2.2)	5.9 (3.6)	5.8 (3.0)	6.1 (5.1)	6.2 (6.1)	6.2 (6.1)	6.2 (6.1)
R_pim_ (%)	0.015 (0.252)	0.015 (0.273)	0.015 (0.388)	0.035 (0.491)	0.039 (0.831)	0.041 (0.816)	0.044 (0.715)	0.049 (0.769)	0.062 (0.790)
CC**_1/2_**	1.000 (0.824)	1.000 (0.790)	1.000 (0.657)	0.999 (0.574)	0.999 (0.368)	0.998 (0.339)	0.998 (0.383)	0.998 (0.806)	0.996 (0.328)
I/σ (*I*)	25.5 (2.7)	25.0 (2.6)	23.8 (1.9)	8.6 (0.9)	7.3 (0.2)	7.1 (0.2)	7.0 (0.2)	6.4 (0.2)	5.4 (0.2)
Completeness (%)	98.1 (84.8)	98.1 (84.7)	98.1 (84.3)	99.9 (98.8)	99.6 (93.7)	99.9 (99.9)	99.9 (99.9)	99.9 (99.9)	99.9 (100)
Wilson B (Å^2^)	11.11	11.38	12.54	8.81	10.57	11.05	11.65	12.70	14.25
Refinement									
Resolution (Å)	1.35	1.35	1.35	1.19	1.22^†^	1.29^†^	1.35^†^	1.45^†^	1.61^†^
Completeness (%)	98.0 (85.4)	98.0 (85.4)	98.0 (85.2)	99.8 (99.0)	96.8 (68.4)^†^	97.3(72.4)^†^	96.6 (67.9)^†^	97.2 (70.1)^†^	96.5 (68.6)^†^
R_work_/R_free_	0.124/0.145 (0.224/0.237)	0.124/0.143 (0.228/0.239)	0.126/0.148 (0.261/0.280)	0.108/0.134 (0.275/0.282)	0.129/0.158 (0.410/0.433)^†^	0.129/0.161 (0.403/0.410)^†^	0.131/0.157 (0.406/0.412)^†^	0.159/0.183 (0.386/0.377)^†^	0.165/0.198 (0.373/0.355)^†^
No. atoms									
Protein	2,790	2,779	2,791	2,814	2,824	2,824	2,824	2,824	2,824
Ligand/ion	51/2	51/2	51/2	145/2	144/2	141/2	141/2	139/2	139/2
Water	621	620	606	553	561	561	563	564	564
B-factors (Å2)									
Protein	14.8	15.0	16.5	14.73	16.23	17.81	19.12	20.82	24.86
Ligand/Cu	35.45/11.99	36.27/12.29	38.99/13.54	35.19/11.48	37.29/12.95	39.69/14.34	40.65/15.66	46.76/16.82	51.11/20.77
Water	27.58	27.99	30.5	30.66	33.02	35.33	36.59	39.67	43.60
R.m.s.^‡^ deviations									
Bond lengths (Å)	0.017	0.017	0.015	0.020	0.018	0.016	0.018	0.013	0.011
Bond angles (°)	2.08	2.09	1.97	2.48	2.35	2.19	2.22	1.94	1.83
PDB ID	7QXK	7QY4	7QYC	7ZCN	7ZCO	7ZCP	7ZCQ	7ZCR	7ZCS

^*^Values in parentheses are for highest-resolution shell.

^†^Resolution cut at refinement stage.

^‡^R.m.s., root-mean-square.

### Intercopper ET in *Br*^2D^NiR Monitored by Laser-Flash Photolysis.

**Fig. 2.**
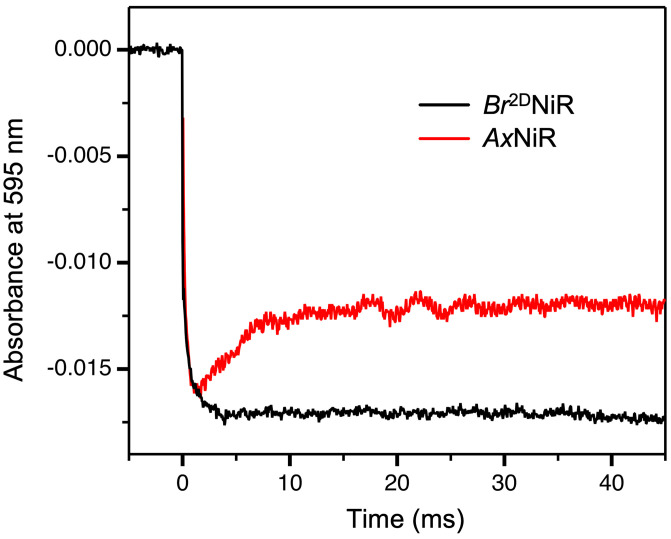
Time-resolved absorbance changes upon laser excitation for as-isolated *Br^2^*^D^NiR and its comparison with as-isolated *Ax*NiR. The initial rapid decrease in absorption at 595 nm is indicative of the reduction of the T1Cu by electrons generated by the photoexcitation of NADH. The slower restorative phase arises from the reoxidation of the T1Cu as ET to the T2Cu occurs. Note that no inter-Cu ET is observed in *Br^2^*^D^NiR (black curve), while recovery of the 595-nm (T1Cu) optical signal is observed in *Ax*NiR, consistent with ET to the T2Cu in *Ax*NiR.

To validate the relevance of MSOX X-ray crystallography data to the solution state, the kinetics of inter-Cu ET from T1Cu to T2Cu in *Br*^2D^NiR was monitored using laser-flash photolysis utilizing the strong charge-transfer band of the Cu^ll^-S_Cys_ of the oxidized T1Cu (13). In these experiments, following the rapid initial reduction of T1Cu, the electron redistributes between the two Cu sites, as a new thermodynamic equilibrium is established, determined by the difference in midpoint reduction potential of the T1 and T2Cu centers. The lack of inter-Cu ET in the absence of nitrite is consistent with the high potential of the T1Cu site in *Br*^2D^NiR (Fig. 2). This is in contrast to *Ax*NiR where the potentials of the two Cu sites are essentially the same and a partial slow recovery of the T1Cu optical signal is observed. In *Br*^2D^NiR,. the T1Cu site potential is ∼50 mV more positive than that of the T2Cu which prevents ET from T1Cu to T2Cu. Despite the presence of the second water ligand in *Br*^2D^NiR, the reduction potential of T2Cu is average for prototypic CuNiRs (25). This lack of ET suggests that in *Br*^2D^NiR, an even stronger gating of ET from T1Cu to T2Cu is in operation in the absence of the substrate. (Table 2 and *SI Appendix*, Fig. S1).

**Table 2.** Redox potentials of *Br*^2D^NiR and *Ax*NiR*

	T1Cu (mV)	T2Cu (mV)
*Br*^2D^NiR	284 ± 11	231 ± 18
*Ax*NiR^‡^	255 ± 3	244 ± 18
*Ax*NiR F306C variant^‡^	262 ± 3	269 ± 11

^*^See *SI Appendix*, Fig. 2 for EPR and optical data used to obtain redox potentials of T2Cu and T1Cu sites, respectively.

^†^Redox values taken from ref. 9.

### MSOX of Nitrite-Soaked *Br*^2D^NiR Single Crystal.

**Fig. 3.**
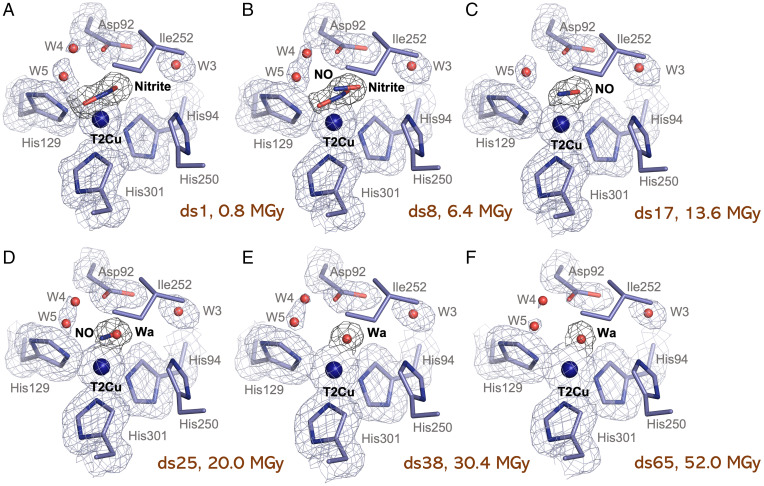
T2Cu site during the MSOX series of a nitrite-soaked *Br*^2D^NiR crystal. (*A*) The T2Cu site after first exposure to 0.8-MGy X-rays (DS1) showing full occupancy of a single side-on nitrite coordinated to T2Cu, Asp92 in proximal position, and two channel waters (W4 and W5). Ile252 and His250 show no changes. (*B*) The T2Cu site in DS8 (6.4 MGy) showing equal occupancies of nitrite and NO. No other changes are seen. (*C*) The T2Cu site in DS17 (13.6 MGy) showing full occupancy of a single side-on NO coordinated to T2Cu. W4 has now disappeared. (*D*) The T2Cu site in DS25 (20 MGy) showing equal occupancies of NO and water (Wa). W4 has now returned. (*E*) The T2Cu site in DS38 (30.4 MGy) showing full occupancy of a single water coordinated to T2Cu, mimicking the oxidized T2CuII site in other prototypic CuNiRs. No other changes are seen. (*F*) The T2Cu site in the final dataset of the nitrite-bound MSOX series (DS65), after a total of 50 MGy, showing the single water (Wa) still coordinated to T2Cu. Asp92 shows signs of burning off due to dose limit in the crystal being exceeded with a loss of density observed. W4 and W5 are also almost completely disappeared. 2Fo − Fc electron density maps of residues are contoured at 1σ level. 2Fo − Fc electron density maps of ligands are contoured at 0.9σ level. T2Cu is shown as a blue sphere.

A 65 dataset MSOX series was also measured at 100K for a single nitrite-soaked *Br*^2D^NiR crystal with a dose of 0.8 MGy per dataset (estimated by RADDOSE-3D(25)) at a resolution ranging from 1.19 Å (DS1) to 1.61 Å (DS65). Data collection and refinement statistics are provided for six structures at various points of the reaction in Table 1 as representatives of the data quality and refinement for the individual structures of the series. In the first dataset (0.8 MGy; 1.19-Å resolution), full occupancy nitrite is seen bound to fully occupied T2Cu in a “side-on” orientation (Fig. 3*A*) with slight asymmetric geometry. Asp92 is seen in a single proximal position with two unique positions of the channel water (W4 and W5) being observed. The catalytic conversion of nitrite begins by dataset 5 (∼4 MGy) and by dataset 8 (6.4 MGy; 1.22-Å resolution), an equal mixture of nitrite and product NO is observed (Fig. 3*B*). After a further 9 datasets (13.6 MGy; 1.29-Å resolution), the conversion of nitrite is completed, and the full occupancy of NO is seen bound to T2Cu in a side-on orientation with near-equidistant ligation of the two atoms (Fig. 3*C*). An intermediate state is observed later in the series as NO starts to be released from the active site and a single water starts to appear with an equal mixture of NO and water seen ligated to T2Cu in dataset 25 (20 MGy; 1.35-Å resolution) (Fig. 3*D*). A single water bound to T2Cu with full occupancy is seen after dataset 38 (30.4 MGy; 1.45-Å resolution) (Fig. 3*E*) as all of the product NO has been released with the active site regaining the water molecule ready for receiving the substrate. The single water forms perfect tetrahedral arrangement and is equivalent to the resting state (T2Cu [Cu^II^]) of other two-domain CuNiRs. The single water remains ligated until the end of the MSOX series (dataset 65) (Fig. 3*F*), similar to results seen for resting state in Fig. 1*C*. In this final dataset (52 MGy; 1.61-Å resolution), X-ray dose exceeded Henderson–Garman limits of ∼40 MGys (26, 27, 28) and signs of “burning off” for the catalytic Asp92 residue are observed, which is consistent with general radiation damage to the structure (Fig. 3*F*). No changes in Ile252 and His250_CAT_ are seen and are the same as in the as-isolated MSOX series. We compared the MSOX series with the MSOX series of the green *Ac*NiR at 100 K (6), the only other case where MSOX has been applied to nitrite bound crystals, to assess the dose dependence of each stage of the catalytic reaction. The conversion of nitrite in *Br*^2D^NiR is significantly slower, and the dose (time) taken to convert all of the substrate to product requires an extra ∼7 MGy of the X-ray dose. This higher dose requirement is also reflected in the dose required before the release of NO from the T2Cu site occurs. The series confirms that the necessary components are present to allow a complete single turnover but at a lower efficiency than *Ac*NiR, consistent with a lower activity of *Br*^2D^NiR. In addition, a reduced tricoordinate T2Cu^I^ site lacking a coordinated water ligand was not observed postreaction as occurred in the MSOX movie of nitrite bound *Ac*NiR at 190K (7).

### Inter-Cu ET in *Br*^2D^NiR in the Presence of the Substrate.

**Fig. 4.**
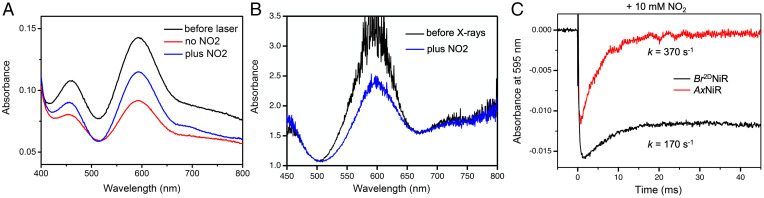
Time-resolved absorbance changes upon laser excitation in the presence of nitrite, absolute absorbance spectra before and after laser excitation, and online optical spectra of single nitrite-bound crystal before and after X-ray exposure used for a dataset for one structure. (*A*) Absolute absorbance spectra of *Br*^2D^NiR in solution in the absence and presence of nitrite observed before and after laser excitation in solution. (*B*) Online optical spectra in a nitrite-soaked crystal of *Br*^2D^NiR before and after X-ray exposure (0.8 MGy). (*C*) Absorbance changes at 595 nm in the presence of excess nitrite (10 mM) observed in the laser flash-photolysis instrument during the reduction of *Br*^2D^NiR and its comparison with equivalent data for *Ax*NiR. Only ∼20% recovery of the 595-nm band is observed in the case *Br*^2D^NiR, indicating a lower degree of ET from T1Cu to T2Cu compared to *Ax*NiR. For fuller experimental details of *Ax*NiR, see ref. 13.

In the presence of excess nitrite (10 mM), the laser-flash photolysis showed that the complete electron redistribution to the T2Cu site does not occur in *Br*^2D^NiR within the 40-ms timescale of the measurements, in contrast to *Ax*NiR where the T1Cu center becomes fully oxidized. The 595-nm band of T1Cu recovers to around a fifth of the initial absorbance in *Br*^2D^NiR, suggesting that only ∼20% electrons are transferred to the T2Cu site compared to complete transfer observed for *Ax*NiR (Fig. 4*A* and *C*). The rate of ET is also slower, namely, 170 s^−1^ compared to 370 s^−1^ with wild-type *Ax*NiR, a rate which is similar to that of the *Ax*NiR variant F306C (9) (in *Br*^2D^NiR, residue 306 is an Ala). The absolute absorbance spectra for *Br*^2D^NiR before and after a laser pulse confirms incomplete inter-Cu ET in the presence of nitrite, consistent with the online optical spectra of a nitrite-soaked crystal taken before and after an ∼0.8-MGy dose of X-rays (Fig. 4*B*). The decreased rate and extent of ET observed for *Br*^2D^NiR in relation to *Ax*NiR are consistent with the relatively lower specific activity of *Br*^2D^NiR. This effect is not due to incomplete metalation of the T2Cu site since the structures show full occupancy of metal and nitrite in the initial structure of the MSOX movie.

To show that the incomplete redistribution of electrons to T2Cu in the presence of nitrite was not due to incomplete substrate binding, steady-state kinetics were measured (*SI Appendix*, Fig. S2). *Br*^2D^NiR revealed an apparent *K*_M_ of 7.4 ± 1.2 µM, a 4-fold increase compared to *Ax*NiR and 160-fold increase when compared to the *Ax*NiR equivalent variant F306C (in *Br*^2D^NiR, F306 is an Ala). Despite this, *Br*^2D^NiR shows 10-fold lower turnover (*k*_cat_ = 9 ± 0.5 s^−1^) and ∼3-fold lower catalytic efficiency when compared to *Ax*NiR but in comparison with *Ax*NiR variant F306C shows a 35-fold lower turnover and some ∼5-fold higher catalytic efficiency (*SI Appendix*, Fig. S2). This suggests that the incomplete redistribution of electrons to T2Cu in *Br*^2D^NiR may arise from factors such as ineffective proton transfer and/or replenishing of the proton source.

## Discussion

Online optical spectroscopy coupled with an MSOX crystallography approach, applied here for the first time, has provided clear evidence that in *Br*^2D^NiR, T1Cu to T2Cu ET does not occur in the absence of nitrite despite a complete X-ray-induced reduction of T1Cu. The absence of inter-Cu ET is also observed in solution being unequivocally confirmed by our laser-flash photolysis experiments. A similar observation was seen in *Ax*NiR single crystals, where on-line X-ray absorption near edge structure (XANES) spectroscopy showed directly that the T2Cu remained oxidized following an X-ray dose that exceeded the threshold for T1Cu reduction (17), establishing a lack of inter-Cu ET in the as-isolated enzyme. However, in the case of *Ax*NiR, laser-flash photolysis experiments had shown a partial reoxidation of the T1Cu center suggesting some ET to the catalytic T2Cu site, which is not the case in *Br*^2D^NiR, providing clear evidence that ET from T1Cu to T2Cu is even more strongly gated in the case of *Br*^2D^NiR, resulting from higher redox potential difference between T1Cu and T2Cu.

The MSOX movie of as-isolated *Br*^2D^NiR together with on-line single crystal spectroscopy is the first application of such an approach. It has provided direct visualization of what happens at the T2Cu site following progressive photoelectron reduction of T1Cu in the absence of nitrite. The five-coordinate T2Cu^II^ oxidized state of *Br*^2D^NiR with two water ligands is converted to a four-coordinate T2Cu^II^ oxidized state with a single water typical of the resting state of prototypic CuNiR. This unique observation suggests that the formation of a three-coordinate T2Cu^(I)^ species that has been shown incapable of binding substrate/ligand in *Ax*NiR (14) is prevented in *Br*^2D^NiR, i.e., *Br*^2D^NiR is well protected from premature reduction of the catalytic site and the formation of a three-coordinate T2Cu dead-end species.

The MSOX serial structures for nitrite-bound *Br*^2D^NiR provide direct evidence for catalysis occurring at the T2Cu site following X-ray-induced reduction of T1Cu, with key intermediates during the catalytic cycle structurally characterized. Of particular note are the observations of side-on bound nitrite and side-on bound NO intermediate prior to returning to a resting state with a single H_2_O ligand to the T2Cu. These structures confirm static damage-free XFEL nitrite-bound and enzymatically produced NO-bound structures in *Br*^2D^NIR (22), with the latter providing further evidence for the formation of a true copper-nitrosyl complex in situ in the crystal during turnover of the substrate. Observation of catalysis at T2Cu contrasts with as-isolated MSOX, as it confirms that inter-Cu ET can occur in the presence of nitrite, with catalysis beginning as early as 5 MGy, confirming that T1Cu-T2Cu ET has happened by this point. A direct comparison with the MSOX series of *Ac*NiR at the same temperature (100 K) (6), shows a higher dose requirement to achieve complete catalytic conversion of nitrite. This is consistent with considerably lower activity of *Br*^2D^NIR compared to both *Ac*NiR and *Ax*NiR (23). MSOX structures of *Br*^2D^NIR show slower conversion of nitrite to NO compared to *Ac*NiR, but also the release of NO from the active site is significantly slower. A noteworthy point is the observation of a single water coordinated to T2Cu postreaction that remains to the end of the MSOX series where the dose of ∼50 MGy has far exceeded the recommended dose limits (25, 27), indicating that the T2Cu site in *Br*^2D^NiR is prevented from the formation of a three-coordinate reduced site. This MSOX movie of nitrite-bound *Br*^2D^NiR together with MSOX serial structures of the as-isolated enzyme clearly demonstrates that the T1Cu-T2Cu ET is strongly gated in *Br*^2D^NiR and occurs with the activation provided by the displacement of coordinated water by the substrate.

Our laser-flash photolysis experiments in the presence of excess nitrite offer a clear reason for lower catalytic inefficiency of *Br*^2D^NiR, as complete ET from T1Cu to the T2Cu site is never achieved in this enzyme with only ∼20% of electrons transferred. The optical spectra before and after laser flash of solution protein and exposure of X-rays to a single crystal with nitrite also confirms this. This is a significant result as it provides a clear explanation for the substantially lower activity seen in *Br*^2D^NiR compared to other classic prototypic CuNiRs such as *Ax*NiR and *Ac*NiR. The single crystal optical data together with MSOX series and steady-state kinetics analysis all point to a rate-limiting proton-coupling event, associated with catalysis which limits the inter-Cu electron redistribution. This together with an unfavorable driving force for ET in the absence of nitrite in *Br*^2D^NiR and severely gated ET results in a slower release of NO from the T2Cu catalytic compared to *Ac*NiR and contributes to the lower activity of *Br*^2D^NiR.

We have shown that a combinational tool of online single crystal spectroscopy with a serial X-ray crystallographic approach like MSOX is powerful for studying catalysis in metalloenzymes, particularly those which utilize a coupled redox system. Given the difficulty in interpreting complex redox reactions, it is clearly a major advantage and advance to couple advanced structural approaches such as MSOX with single crystal spectroscopy alongside solution data. The spectroscopically validated serial structures provide a clean structural context for understanding reactions in solution, monitored by ensemble spectroscopy/kinetic methods.

## Materials and Methods

### Protein Expression, Purification, and Crystallization.

Expression of recombinant *Br*^2D^NiR (with 6xHis tag) into *Escherichia coli* was performed as previously described (22, 23). For purification, a cell pellet, resuspended in 100 mM Tris-HCl and 500 mM NaCl buffer (pH 8.0) (buffer A) and supplemented with EDTA-free protease inhibitor mixture (Roche), was disrupted with lysozyme (50 µg/mL) and sonication. All steps after this were performed at 4 °C. The cell lysate, collected by centrifugation, was loaded onto a HisTrap high performance Ni affinity column (GE Healthcare) and equilibrated with 10 mM imidazole in buffer A. Bound protein was eluted with 250 mM imidazole in buffer A and dialyzed overnight against 100 mM Tris-HCl, 500 mM NaCl, and 10% (vol/vol) glycerol (pH 8) (buffer B). The 6xHis tag was cleaved by overnight digestion with TEV (Tobacco Etch Virus) protease and the addition of 2 mM dithiothreitol (DTT) and then loaded onto a HiLoad Superdex 200 16/600-pg size exclusion chromatography (SEC) column (GE Healthcare) equilibrated with buffer B. Protein was dialyzed overnight against 1 mM CuSO_4_ in buffer B and reran on an SEC column. Full copper loading of the T2Cu site was achieved by a final overnight dialysis against 1 mM CuSO_4_ in buffer B before a final run on the SEC column. Protein was concentrated to final concentration of ∼30 mg/mL and before crystallization was buffer exchanged into 10 mM Hepes (pH 6.5).

Crystals of *Br*^2D^NiR were grown using the vapor diffusion hanging drop method at room temperature and by mixing protein with 30 mg/mL concentration with reservoir solution in a 1:1 ratio. The reservoir solution composed of 1.8 M ammonium sulfate with 50 mM Hepes buffer (pH 5). Crystals in space group *P*2_1_3 grew within a few days.

### MSOX and On-Line Single Crystal Spectroscopy of *Br*^2D^NiR in As-Isolated State.

For MSOX of a single *Br*^2D^NiR crystal in an as-isolated state, a 0.4- × 0.4- × 0.4-mm crystal was soaked in a cryoprotectant solution composed of 3.3 M ammonium sulfate, 20.3% sucrose, and 50 mM Hepes buffer (pH 5.5) before being cryocooled by plunging into liquid nitrogen. The crystal was kept at 100 K on beamline BL26B1 at SPring-8. Online single crystal optical spectrum was measured before X-ray exposure using a fiberoptic microspectrophotometer with a linear charge-coupled device array detector (Ocean Insight, SD2000) using a halogen light source (Ocean Insight, HL-2000-HP-FHSA), with wavelength limited to the visible light region. Crystallographic data were collected using a DECTRIS EIGER4M detector at an X-ray wavelength of 0.99999 Å, with flux beam intensity calculated as 1.13 × 10^11^ photon/s and max total dose per dataset calculated as 0.4 MGy using RADDOSE-3D (25). The same exposed volume of the *Br*^2D^NiR crystal was used for 20 consecutive, complete crystallographic datasets with online optical spectrum measured after each dataset, limited to visible light wavelength region. Data were processed using X-ray detector software) (29) and the automated data processing pipeline KAMO at SPring-8 (30). Refinement was performed using Refmac5 (31) in the CCP4 suite (32) with manual rebuilding in Coot (33) and isotropic B-factors.

### MSOX of a Single Nitrite-Soaked *Br*^2D^NiR Crystal.

A single *Br*^2D^NiR crystal was soaked into a nitrite-containing a solution composed of 200 mM NaNO_2_, 2.5 M ammonium sulfate, and 50 mM Hepes buffer (pH 5.5) before being transferred into a cryoprotectant solution composed of 200 mM NaNO2, 3.3 M ammonium sulfate, 20.3% sucrose, and 50 mM Hepes buffer (pH 5.5) and cryocooled by plunging into liquid nitrogen. Crystallographic data were collected using a DECTRIS Pilatus36M detector at beamline I24 at DIAMOND Light Source at an X-ray wavelength of 0.99999 Å, with flux beam intensity calculated as 6.00 × 10^11^ photon/s and max total dose per dataset calculated as 0.8 MGy using RADDOSE-3D (25). The same exposed volume of the *Br*^2D^NiR crystal was used for 65 repeated, complete crystallographic datasets. Data were processed using DIALS (34) and xia2 (35) through the automated data processing pipeline at DIAMOND. Due to low completeness in the last shell, DS1 was rescaled in AIMLESS (34) to a resolution of 1.19 Å. Refinement was performed using Refmac5 (29) in the CCP4 suite (30) with resolution for DS8, DS17, DS25, DS38, and DS65 cut manually in Refmac5 in order to improve last shell refinement completeness of data. Manual rebuilding was performed in Coot (33) with anisotropic B-factors up to a resolution of 1.35 Å and isotropic B-factors thereafter.

### Laser-Flash Photolysis.

Laser-flash measurements were performed at 4 °C using an approach that was previously described (36). Samples containing CuNiR (approx. 30 μM), NADH (200 μM), and NMN (50 mM) were excited at 355 nm using the third harmonic of a Q-switch Nd:YAG laser (Brilliant B, Quantel), and time-resolved spectral changes associated with the Cu^II^ state of the T1Cu (at 600 nm) were used to monitor rates of ET. Acquired transients were fitted to exponential decay functions using OriginPro 9.1.

### Redox Potentiometry Measurements and Electron Paramagnetic Resonance.

The reduction potentials of the T1 and T2Cu centers in *Br*^2D^NiR were determined by titration with sodium dithionite. To facilitate communication between the electrode and the protein, redox mediators were used. The electrochemical potential of the solution was measured using a Thermo Orion oxygen reduction potential electrode at 25 °C. A factor of +207 mV was used to correct values to the standard hydrogen electrode. During titration against dithionite, samples were withdrawn for electron paramagnetic resonance (EPR) analysis. They were placed in 4-mm OD Suprasil quartz EPR tubes (Wilmad LabGlass) under anaerobic conditions and immediately frozen in liquid N_2_. To determine the redox potentials of the two copper centers, EPR spectroscopy was used. Continuous wave X-band EPR spectra (∼9.4 GHz) were recorded using a Bruker ELEXSYS E580 EPR spectrometer (Bruker GmbH) using a super-high-Q resonator. Temperature was maintained using an Oxford Instruments ESR900 helium flow cryostat coupled to an ITC 503 controller from the same manufacturer. EPR experiments were carried out at 20 K and employed 0.5-mW microwave power, 100-kHz modulation frequency, and 5-G (0.5 mT) modulation amplitude. Redox potentials of the copper centers were determined by fitting data to the Nernst equation.

## Supplementary Material

Supplementary File

Supplementary File

## Data Availability

The atomic coordinates have been deposited in the Protein Data Bank (PDB identifier [ID] codes: 7QXK, 7QY4, 7QYC, 7ZCN, 7ZCO, 7ZCP, 7ZCQ, 7ZCR, and 7ZCS (37–45)). All study data are included in the article and/or *SI Appendix*.
