# Supplementary material for: Single crystal spectroscopy and multiple structures from one crystal (MSOX) define catalysis in copper nitrite reductases
Source: Proc Natl Acad Sci U S A. 2022 Jul 21;119(30):e2205664119. doi: 10.1073/pnas.2205664119 (PMC9335323; doi:10.1073/pnas.2205664119)

Single crystal spectroscopy and multiple structures from one crystal (MSOX) define catalysis in copper Nitrite Reductases

Samuel L. Rose[1], Seiki Baba[2], Hideo Okumura[2], Svetlana V. Antonyuk[1], Daisuke Sasaki[1†], Tobias M. Hedison[4], Muralidharan Shanmugam[4], Derren J. Heyes[4], Nigel S. Scrutton[4], Takashi Kumasaka[2], Takehiko Tosha[3], Robert R. Eady[1], Masaki Yamamoto[3], S. Samar Hasnain[1*]

[1]Molecular Biophysics Group, Life Sciences Building, Institute of Systems, Molecular and Integrative Biology, Faculty of Health and Life Sciences, University of Liverpool, L69 7ZB, UK; [2]Protein Crystal Analysis Division, Japan Synchrotron Radiation Research Institute, 1-1-1 Kouto, Sayo-cho, Sayo-gun, Hyogo, 679-5198, Japan; [3]RIKEN SPring-8 Center, 1-1-1 Kouto, Sayo, Hyogo, 679-5148, Japan; [4]Manchester Institute of Biotechnology and School of Chemistry, Faculty of Science and Engineering, The University of Manchester, 131 Princess Street, Manchester M1 7DN, United Kingdom

[†]Current address: Pharmaceutical Chemistry Laboratory, Faculty of Pharmaceutical Sciences, Wakayama Medical University, Japan.

* Corresponding Author. Email: s.s.hasnain@liverpool.ac.uk

Supplementary figures

Supplementary Figure 1. Determination of the redox potential of *Br*[2D]NiR by electron paramagnetic resonance spectroscopy. A) The continuous wave electron paramagnetic resonance spectra of *Br*[2D]NiR during reduction with sodium dithionite. B) Fraction of the T1 and T2Cu sites oxidised as a function of redox potential. Data in B are fit to the Nernst equation.

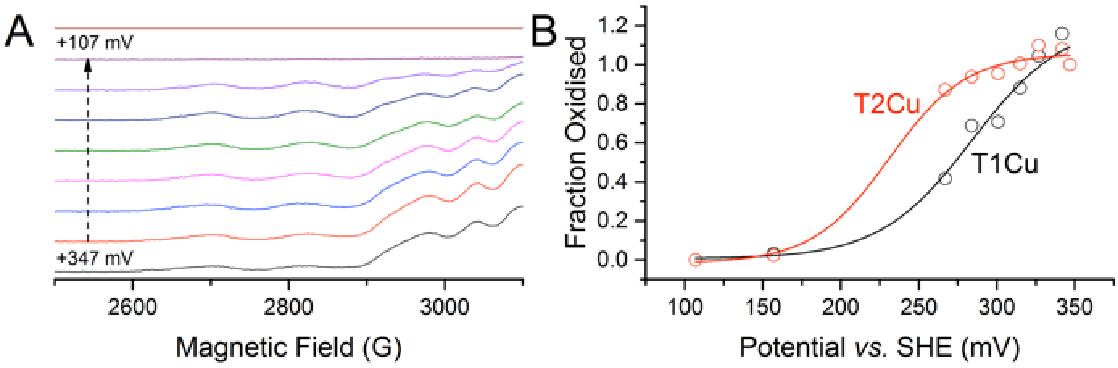

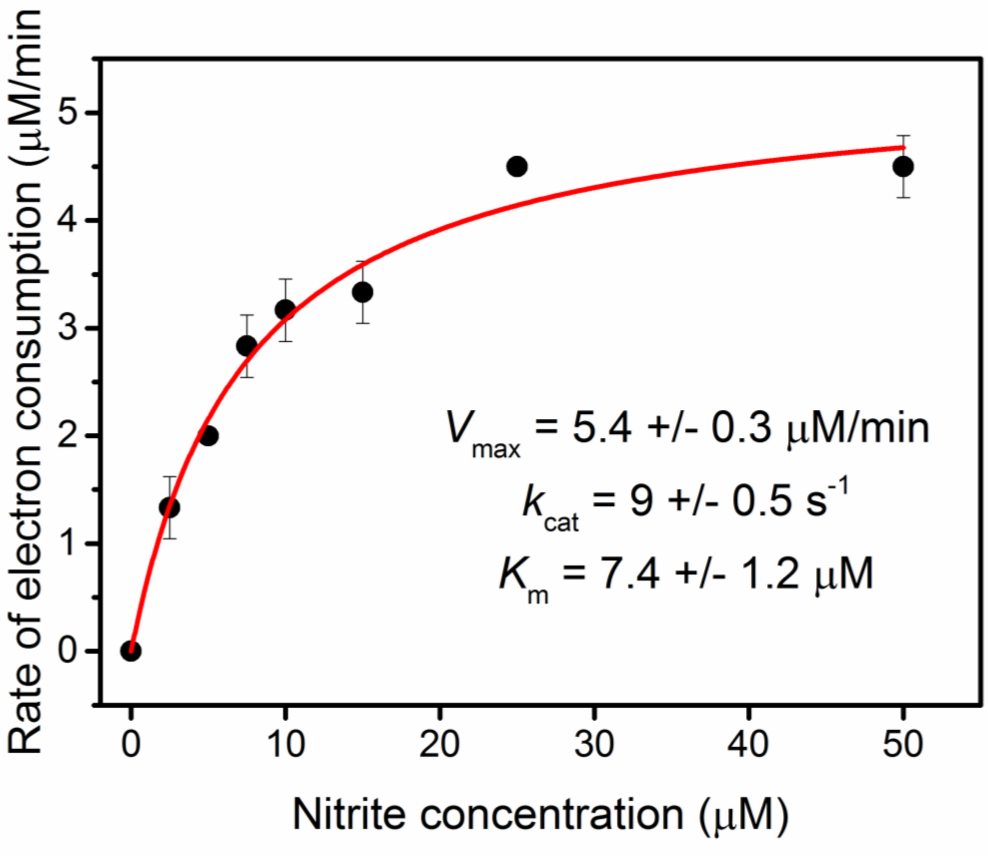

Supplementary Figure 2. Steady-state kinetics of *Br*^{2D}NiR. To monitor the conversion of nitrite to nitric oxide, the electron donor sodium dithionite was measured at 315 nm. Assays contained catalytic amounts of *Br*^{2D}NiR, 0.5 mM of sodium dithionite and 0.1 mM phenazine methylsulfate (PMS) as a mediator.

Movie 1, supplementary data

MSOX movie of the as-isolated *Br*^{2D}NiR enzyme.

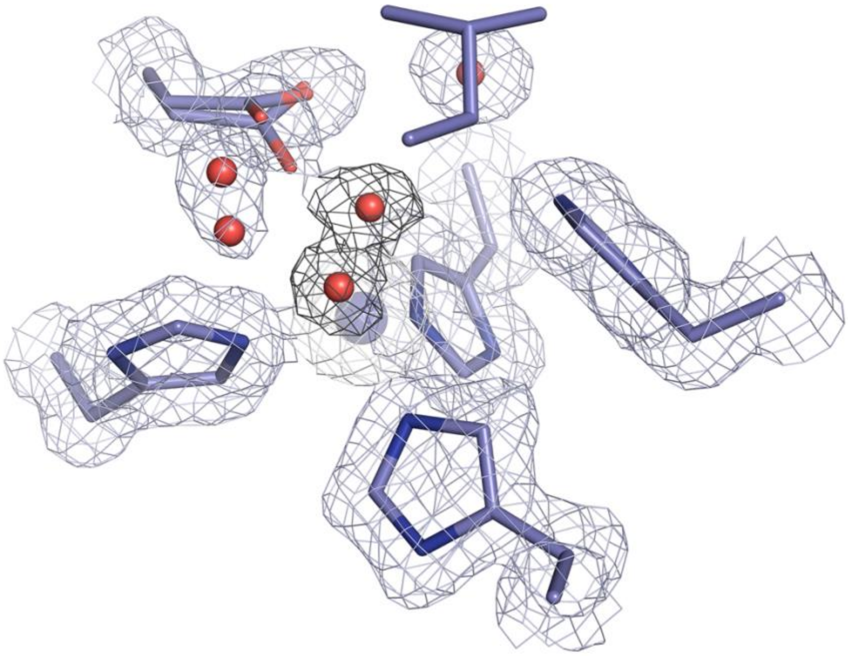

Movie 2, supplementary data

MSOX movie of the nitrite bound *Br*^{2D}NiR enzyme.

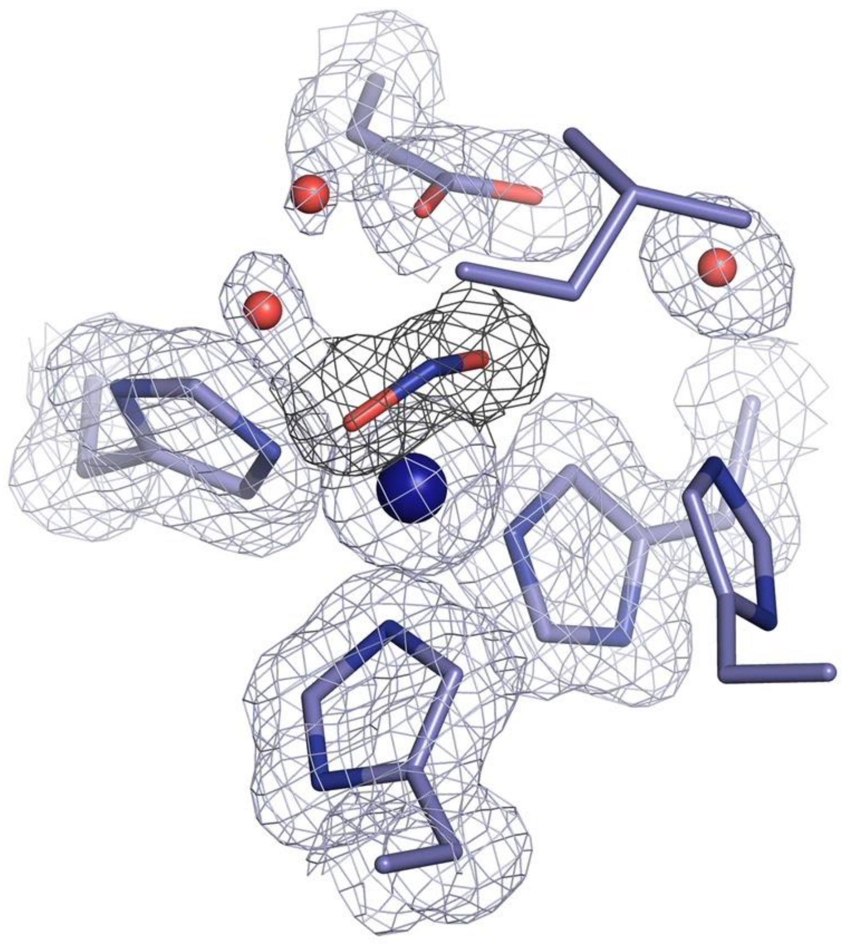

Supplement: Supplementary File [file pnas.2205664119.sapp.pdf]
